# TSG: a new algorithm for binary and multi-class cancer classification and informative genes selection

**DOI:** 10.1186/1755-8794-6-S1-S3

**Published:** 2013-01-23

**Authors:** Haiyan Wang, Hongyan Zhang, Zhijun Dai, Ming-shun Chen, Zheming Yuan

**Affiliations:** 1 Department of Statistics, Kansas State University, Manhattan, KS 66506, USA; this work was done while Haiyan Wang was on sabbatical leave at Hunan Provincial Key Laboratory of Crop Germplasm Innovation and Utilization, Changsha 410128, China; 2 Hunan Provincial Key Laboratory of Crop Germplasm Innovation and Utilization, Changsha 410128, China; 3 College of Information Science and Technology, Hunan Agricultural University, Changsha 410128, China; 4 College of Bio-safety Science and Technology, Hunan Agricultural University, Changsha 410128, China; 5 USDA-ARS and Department of Entomology, Kansas State University, Manhattan, KS 66506, USA

## Abstract

**Background:**

One of the challenges in classification of cancer tissue samples based on gene expression data is to establish an effective method that can select a parsimonious set of informative genes. The Top Scoring Pair (TSP), k-Top Scoring Pairs (k-TSP), Support Vector Machines (SVM), and prediction analysis of microarrays (PAM) are four popular classifiers that have comparable performance on multiple cancer datasets. SVM and PAM tend to use a large number of genes and TSP, k-TSP always use even number of genes. In addition, the selection of distinct gene pairs in k-TSP simply combined the pairs of top ranking genes without considering the fact that the gene set with best discrimination power may not be the combined pairs. The k-TSP algorithm also needs the user to specify an upper bound for the number of gene pairs. Here we introduce a computational algorithm to address the problems. The algorithm is named Chisquare-statistic-based Top Scoring Genes (Chi-TSG) classifier simplified as TSG.

**Results:**

The TSG classifier starts with the top two genes and sequentially adds additional gene into the candidate gene set to perform informative gene selection. The algorithm automatically reports the total number of informative genes selected with cross validation. We provide the algorithm for both binary and multi-class cancer classification. The algorithm was applied to 9 binary and 10 multi-class gene expression datasets involving human cancers. The TSG classifier outperforms TSP family classifiers by a big margin in most of the 19 datasets. In addition to improved accuracy, our classifier shares all the advantages of the TSP family classifiers including easy interpretation, invariant to monotone transformation, often selects a small number of informative genes allowing follow-up studies, resistant to sampling variations due to within sample operations.

**Conclusions:**

Redefining the scores for gene set and the classification rules in TSP family classifiers by incorporating the sample size information can lead to better selection of informative genes and classification accuracy. The resulting TSG classifier offers a useful tool for cancer classification based on numerical molecular data.

## Background

With the availability of high throughput genomics data, methods for cancer class classification and prediction based on molecular information have been vigorously pursued in recent years. The objective of this study is to find important molecular markers and/or build a classifier such that the classifier with selected markers as the independent variables can accurately classify the diagnostic disease status of a sample using expression data. Popular methods for this problem include Prediction Analysis of Microarrays (PAM, [1]), Top Scoring Pair (TSP, [2]), k-Top Scoring Pair (k-TSP, [3]), Support Vector Machine (SVM, [4]) etc. There are also many other endeavors such as individual-gene-ranking by evaluating the discriminating power of classes (see [5,6] and the references therein), gene filtering through relevance and correlation analyses [7,8], gene selection for classification based on the Bayes error [9], comparing the distributions of within-class correlations with between-class correlations via Kullback-Leibler divergence [10], recursive feature addition with Lagging Prediction Peephole Optimization to choose the final optimal marker set [11], SVM based recursive feature elimination [12,13], random forests [14] and random subspace search [15,16], among others.

There are a few challenges associated with such study. One of them is that the number of independent variables (markers) is typically much more than the number of available samples, often referred as curse of dimensionality. To identify possibly nonlinear effects of many variables and their interactions, it is often necessary to estimate a large number of modeling parameters. A direct consequence of the curse of dimensionality is that the total number of parameters that the data can estimate is restricted by the number of the samples. When the total number of parameters greatly exceeds the number of samples, overfitting occurs such that the prediction of the phenotype works well for the learning data but the performance of the classifier applied to independent test samples exhibit poor classification accuracy. The informative marker selection process unfortunately needs to consider modeling with each possible combination of all markers in order to find the globally best marker set, which has the best discriminating power for the different disease categories and may or may not be the primary biological and pathological driving factors underlying disease progression. Hence, an effective practice is to first reduce the dimensionality of the marker space.

The TSP and k-TSP classifiers are two simple algorithms that select gene pairs with top scores to build classifiers. They were shown to perform well for binary classification with gene expression data [2,3]. The gene pairs were selected based on simple pairwise comparisons between two marker expression levels within the same sample. Specifically, let *p_ij_(C_1_) *be the percentage of training samples in class 1 that the expression of one marker is less than that of the other marker in the same sample and let *p_ij_(C_2_) *be similarly defined. The score for a gene pair is defined as the estimated difference between the two percentages *p_ij_(C_1_) - p_ij_(C_2_)*. Then the gene pair that received the highest score is selected as the marker set for TSP classifier and the top k gene pairs with highest scores are used for the k-TSP classifier. Tan et al. [3] extended the two classifiers to multi-class classification through one-vs-others, one-vs-one, and hierarchical classification (HC) schemes. They reported that the HC schemes for TSP and k-TSP gave better performance than the other two schemes.

There are advantages and disadvantages with the TSP and k-TSP classifiers. Some advantages of the two classifiers are that they are simple to implement and the resulting classifiers are easy to interpret. They are also invariant to monotone transformations as they only depend on relative rankings of gene expressions within the same sample. The overfitting problem is largely avoided due to simple comparisons. In addition, they are different from most algorithms in that comparisons in other algorithms were mostly between expressions from different samples. Comparison of expressions within the same sample in TSP and k-TSP helps to eliminate the influence of sampling variability due to different subjects.

A disadvantage is related to how the scores for gene pairs are defined. As the scores were calculated from percentages, the sample size information was not fully utilized in TSP and k-TSP. For example, suppose 4 out of 10 samples in class 1 and 6 out of 10 samples in class 2 satisfy the condition: Marker 1 has smaller expression value than marker 2. The score for the pair with markers 1 and 2 is 0.2, which is the absolute difference between the two percentages. In another case, suppose all the counts are multiplied by 10, i.e. 40 out of 100 samples in class 1 and 60 out of 100 samples in class 2 satisfy the condition. Then the score for the marker pair is identical to the previous case. So the additional information with extra sample size is completely ignored in TSP and k-TSP classifiers.

The multi-class classifiers HC-TSP and HC-k-TSP are two versions that showed best performance among all TSP family classifiers [3]. They were derived from a scheme that performs sequential binary classification. At the root node, the training samples are partitioned into two classes, the largest class and the composite class. The largest class containing the largest number of samples is treated as a leaf node for final classification of the phenotype. The composite class is then further partitioned similarly as the root partition. This scheme intends to balance the two classes during each binary partition. However, the markers selected at each binary partition with TSP or k-TSP are not necessarily the best marker set to separate all the classes since they are selected based on their differentiating ability to separate the largest class from the composite class at the node. In addition, the selection of markers at each partition does not have a mechanism to control the redundancy of the candidate marker set. For example, in prostate cancer LNCaP cells, forkhead transcription factor (FOXO3a) that is the phosphatidylinositol 3-kinase (PI3K/Akt) downstream substrate, is a positive regulator for the induction of androgen receptor (AR) gene expression. The blocking of AR functions by AR interfering RNA leads to dramatic LNCaP cell death. Hence the inhibition of the PI3K/Akt pathway may result in the activation of the FOXO3a transcription factor, which may then induce the AR gene expression to protect cells from apoptosis of LNCaP prostate cancer cells. The PI3K/Akt and FOXO3a could both be selected in the marker selection algorithm of HC-TSP or HC-k-TSP. Apparently, they are highly correlated.

In this article, we propose a new algorithm to overcome the above problems of TSP family classifiers. We introduce a new definition of the score for each marker set so that the sample size information is fully utilized. In addition, it is unrealistic to assume that the number of informative genes is always even as in TSP family classifiers. We present a new algorithm that performs sequential search and do not restrict the informative markers to be even numbered. The binary class and multi-class cases are unified into a single framework. The algorithm was applied to 9 binary class and 10 multi-class cancer genomics datasets. The TSG classifier achieved better leave-one-out cross validation accuracy for the binary classification than TSP or k-TSP classifiers. For the multi-class problems, our TSG classifier gives comparable performance or outperform TSP family and other popular classifiers with a big margin in independent test accuracy for several cancer datasets. Beyond high accuracy, our new algorithm also has the advantage of giving small number of informative marker set and all the advantages of the TSP family classifiers.

## Methods

For generality, we describe the method in terms of markers, which could represent genes, probe sets, or other molecular units whose intensity is measured with high throughput instruments. Consider expression data from *P *markers and suppose there are *N *samples. The data can be expressed as a matrix ***X ***of dimension *N*x*P*. The (*i, j*) element *x_ij _*of the matrix gives the expression value of the j^th ^marker in the *i^th ^*sample. Let (*y_1_,..., y_N_*) be the class labels for the N samples, where *y_k _*takes one of the values in the set of all possible classes {*C_1_,..., C_M _*}. C_i _represents the class phenotype that may be cancerous tumor, normal, or cancer subclasses such as different stages of a cancer. Denote *x_i _= *(*x_i1_,..., x_iP_*) to be the *P *expression values from the *i^th ^*sample. The *P *is typically much larger than *N*, and could be in the neighborhood of exponential order O(*e^N^*) with high density microarrays. The objective is to use Ω = { (*x_i _, y_i_*), *i = 1,..., N*} to select a parsimonious set of informative markers and build a classifier with these markers such that the diagnosis status of a test sample can be accurately classified by modeling the expression data of the selected markers.

### Score of a marker set

To consider the differentiating power of a set of markers consisting of k markers, we first define the score of the marker set. A normal sample contains normal proto-oncogenes that promote cell growth and mitosis and tumor suppressor genes that discourage cell growth. During cancer development, proto-oncogenes can be mutated by carcinogenic agents to become oncogenes, which produce excessive levels of growth promoting proteins. Cancer results from cumulative mutations of proto-oncogenes and suppressor genes which together allow the unregulated growth of cells. Hence, cancer development involves uncontrolled cell division resulting from a series (progression) of gene mutations that typically involve two categories of function: promotion of cell division and inactivation of cell cycle suppression. The expression values of excessive growth genes tend to be much higher than the same genes of a normal sample. Similarly, the level of tumor suppressor genes of a cancer sample tends to be much lower than a normal sample. It is relatively rare that a single marker alone could offer sufficient power to differentiate all the classes well in multi-class data. So we consider marker sets with at least two markers keeping in mind that over-growth of some genes and inactivation of other genes often happen together in cancer cases. Later stages of cancer involve tissue invasiveness, during which malignant cells travel among tissues via the circulatory and/or lymphatic system and grow and thrive in their new locations. Therefore the relative amount of two or multiple markers in a sample could be an indication of the cancer stages.

### Score of marker pairs

For markers *i *and *j*, we use the following notation. Let *f*_1mij _, *m *= 1,... *M*, represent the frequency count of samples in class *C_m _*that satisfy the condition: the expression value for marker *i *is less than the expression value of marker *j*. Similarly, let *f*_2mij _, *m *= 1,... *M*, be the frequency count of samples that satisfy the condition: the expression value for marker *i *is greater than or equal to the expression value of marker *j*. These counts can be presented in a cross-tabulation table as shown in Table 1, where *f*_1mij _, m = 1,... M, are the entries in the first column and *f*_2mij _, m = 1,... M, are the entries in the second column.

**Table 1:** Frequency counts of samples in each class for marker pairs.

	*E*_i _< E_j_	*E*_i _≥ *E*_j_	Total
**Class *C*_1_**	*f*_11ij_	*f*_21ij_	***n*_1 _= *f*_11ij _*+f*_21ij_**
⋮	⋮	⋮	⋮
**Class *C*_M_**	*f*_1Mij_	*f*_2Mij_	***n*_M _=*f*_1Mij _*+f*_2Mij_**
	
**Total**	$T_{1}=\Sigma_{m=1}^{M}f_{1mij}$	$T_{2}=\Sigma_{m=1}^{M}f_{2mij}$	***N***

The *E_i _*and *E_j _*represent the population of expression values for markers i and j respectively.

The n_i _are the total number of samples in class *C*_i_, *i = 1,..., M*.

Based on the cancer mechanism that there is excessive growth in tumor cells and inactivation of suppressor genes, the best informative genes would consist of some genes overly expressed and some other genes that are down-regulated. In particular, a marker pair with genes i and j become increasingly more informative of the cancer status as the difference of their expression values diverges away from the corresponding difference between the same marker pairs of a normal patient. Consequently, for two markers encoding genes or proteins that are important to differentiate the cancer status, their relative magnitude of the expressions are inter-related and whether the expression value for marker *i *is less than the expression value of marker *j *is not independent of the class status. To incorporate the sample size information, the Chisquare statistic defined in equation (1) can be used to assess whether the pair of markers i and j are informative for classification of cancer status:

(1) $${\chi_{ij}}^{2}=\left(\Sigma_{q=1}^{2}\Sigma_{m=1}^{M}\frac{{\left(f_{qmij}-n_{m}T_{q}/N\right)}^{2}}{n_{m}T_{q}/N}\right)=N\left(\Sigma_{q=1}^{2}\Sigma_{m=1}^{M}\frac{f_{qmij}^{2}}{n_{m}T_{q}}-1\right),$$

where *n_m _*and *T_q _*are the row and column totals from the *m^th ^*row and *q^th ^*column, respectively. If all the counts in Table 1 are large and all cell counts are at least five, a traditional way to declare significance for the pair is to compare the calculated statistic with the chi-squared-distribution with *M -*1 degrees of freedom. However, the significance level for declaring significance of a single test needs to be adjusted for multiple comparisons. There are various directions including family-wise error rate control, false-discovery rate (FDR) control, among others. The family-wise error rate control tends to be conservative while the FDR control could lead to high false positive rates. In this work, we do not use the chi-squared-distribution and do not decide how many pairs are significant. Instead, we only use the Chi-square statistic in (1) as an indication of how much departure from independence between the class and the chance of observing marker i expression value less than that of marker j. As the departure from independence increases, the chi-squared statistic value increases. We select the top pair of markers that yield the highest value of the chi-squared statistic. Additional marker selection will follow the algorithm in section 2.2.

### Score of a *k*-marker set

For a set that contains k markers, we consider the cross tabulated Table 2 that contains frequency counts of all unique pairwise comparisons among the *k *markers. There are *k*(*k-1*) columns of counts. The sum of counts in each column remains to be the same as that for two-marker case. The row totals are now *k *times that of the sample sizes in corresponding classes. We calculate the Chisqure statistic as in equation (2).

**Table 2:** Frequency counts of samples in each class for a set of k markers.

Class	$E_{i_{1}}<E_{i_{2}}$	$E_{i_{1}}\geq E_{i_{2}}$	...	$E_{i_{k-1}}<E_{i_{k}}$	$E_{i_{k-1}}\geq E_{i_{k}}$	Total
***C*_1_**	$f_{11i_{1}i_{2}}$	$f_{21i_{1}i_{2}}$	...	$f_{11i_{k-1}i_{k}}$	$f_{21i_{k-1}i_{k}}$	***kn_1_***
⋮	⋮	⋮	⋮	⋮	⋮	⋮
***C*_M_**	$f_{1Mi_{1}i_{2}}$	$f_{2Mi_{1}i_{2}}$	...	$f_{1Mi_{k-1}i_{k}}$	$f_{2Mi_{k-1}i_{k}}$	***kn_M_***
	
**Total**	$T_{1}=\Sigma_{m=1}^{M}f_{1mi_{1}i_{2}}$	$T_{2}=\Sigma_{m=1}^{M}f_{2mi_{1}i_{2}}$	**...**	$T_{\left(\text{k}-1\right)\text{k}/2-1}=\Sigma_{m=1}^{M}f_{1mi_{1}i_{2}}$	$T_{\left(\text{k}-1\right)\text{k}/2}=\Sigma_{m=1}^{M}f_{2mi_{1}i_{2}}$	***K****N*

(2) $${\chi^{2}}_{i_{1}...i_{k}}=\Sigma_{a=1}^{k-1}\Sigma_{b=a+1}^{k}\Sigma_{m=1}^{M}\Sigma_{q=1}^{2}\frac{{\left(f_{qmi_{a}i_{b}}-\frac{kn_{m}T_{q}}{N}\right)}^{2}}{kn_{m}T_{q}/N}=N\left(\Sigma_{a=1}^{k-1}\Sigma_{b=a+1}^{k}\Sigma_{m=1}^{M}\Sigma_{q=1}^{2}\frac{f_{qmi_{a}i_{b}}^{2}}{kn_{m}T_{q}}-1\right),$$

Note that the ${\chi^{2}}_{i_{1}...i_{k}}$only differs from $\Sigma_{a=1}^{k-1}\Sigma_{b=a+1}^{k}{\chi^{2}}_{i_{a}i_{b}}$by the division factor *k *in the first term. So comparison of ${\chi^{2}}_{i_{1}...i_{k}}$ and ${\chi^{2}}_{j_{1}...j_{k}}$ for two sets of *k*-markers {*i_1_,..., i_k_*} and {*j_1_,..., j_k_*} is equivalent to comparing $\Sigma_{a=1}^{k-1}\Sigma_{b=a+1}^{k}{\chi^{2}}_{i_{a}i_{b}}$and $\Sigma_{a=1}^{k-1}\Sigma_{b=a+1}^{k}{\chi^{2}}_{j_{a}j_{b}}$. The latter can be calculated easily without much computational cost after the statistics for marker pairs have been computed. The statistics given in equation (2) should be restricted to comparing marker sets with the same number of markers. All the k-marker sets can be ranked according to the magnitude of the Chisquare statistic values. The k-marker set with the highest Chisquare value is the most informative set among all k-marker sets.

### Comparing marker sets of different sizes or identical Chisquare statistic

For comparing multiple sets with different numbers of markers, the Chisquare statistics given earlier can not be used because they accumulate different numbers of terms. In such case, we use the leave-one-out cross validation (LOOCV) accuracy within the training data obtained with the procedure below as the objective function.

Suppose the training data contains *N_tr _*samples. Without loss of generality, we use Ω_tr _= {*S_1_*,..., *S_Ntr_*} to denote the collection of these samples. For a marker set {*i_1_,..., i_k_*}, the LOOCV is performed within this training sample. In particular, we

1. Leave out one training sample *S_l _*to be used as the test data and use the rest of the training samples Ω_tr_\ *S_l _*as training data.

2. For class *m*, (*1 *≤ *m *≤ *M*), assign *S_l _*to this class and calculate the Chisquare statistic for the marker set {*i_1_,..., i_k_*}. We obtain M Chisquare statistics {${\chi^{2\left(m\right)}}_{i_{1}...i_{k}}$, *m = 1,..., M*}. The predicted class $\hat{m}\left(S_{l}\right)$for *S_l _*is the class that has the maximum Chisquare statistics, i.e., $\hat{m}\left(S_{l}\right)=\text{arg}\;\underset{1\leq m\leq M}{\text{max}}{\chi^{2\left(m\right)}}_{i_{1}...i_{k}}$.

3. Repeat 1 and 2 for all the samples in Ω_tr _to get the prediction of all samples in Ω_tr_: $\hat{m}\left(S_{l}\right)$, *l *= *1*,..., *N_tr_*.

4. The LOOCV accuracy for marker set {*i_1_,..., i_k_*} is LOOCV(*i_1_,..., i_k_*) = the proportion of correctly classified samples in Ω_tr_.

The comparison of marker sets of different sizes can be based on the LOOCV accuracy within the training data. The marker set with highest LOOCV is more informative than other marker sets. For marker sets with the same number of markers and identical Chisquare statistic value, we also use the LOOCV accuracy as a measure of their differentiating power toward the different cancer classes.

### Marker selection algorithm

For a given upper bound *B *on the cardinality of the marker set, the marker selection process first selects the top scoring pairs and then sequentially adds additional markers into the active set until the total number of markers in the active set reaches the upper bound *B*. This is done following the algorithm below: Denote the set of remaining markers as ẞ. The initial value of ẞ is the list of all markers.

1. Calculate and record the Chisquare statistics for all marker pairs using the training data.

2. If the highest value of the Chisquare statistics is achieved by a unique marker pair, select this pair and denote it as TS_2_. Calculate the LOOCV for this pair of markers and denote it as LOOCV_2_. Update the remaining marker set ẞ by removing the marker pair selected.

3. If there are multiple marker pairs that have identical maximum Chisquare statistic value, calculate the LOOCV accuracy of these marker pairs using the training data. Keep the marker pairs that have the highest LOOCV accuracy. If the highest accuracy is achieved by more than one pairs, denote the different pairs as TS_2,1_, TS_2,2_, etc.

4. Find the top scoring triplets by adding additional marker to the top scoring pairs. This is done as follows. For each of the top scoring pairs resulting from 2 and 3, find the marker from the list of remaining markers ẞ such that the triplet has the highest Chisquare statistic value. If there are multiple triplets with identical maximum Chisquare value, calculate the LOOCV accuracy of these triplets and record those triplets that yield the highest LOOCV accuracy. Denote the top scoring triplet as TS_3 _if it is unique, and TS_3,1_, TS_3,2_, etc otherwise if multiple sets achieved identical accuracy. Record the LOOCV accuracy of the top scoring triplets.

5. For *k *= *4, 5*,..., *B*, find TS_k _and their corresponding LOOCV accuracy LOOCV_k_. As *k *increases, the set TS_k _tend to be unique.

6. Select the smallest k-marker set such that the LOOCV is maximized over all TS_k_, *k = 1*,..., *B*. If the marker set is not unique, randomly select one of them as the final set. Denote the final selected informative marker set as TS_G_, where G = arg max_1 ≤ k ≤ B _LOOCV_k_.

As discussed in section 2.1.2, the comparison of the Chisquare statistics for ${\chi^{2}}_{i_{1}...i_{k}}$can be simplified by comparing the summation of all Chisquare statistics from unique marker pairs $\Sigma_{a=1}^{k-1}\Sigma_{b=a+1}^{k}{\chi^{2}}_{i_{a}i_{b}}$.

**Figure 1:**
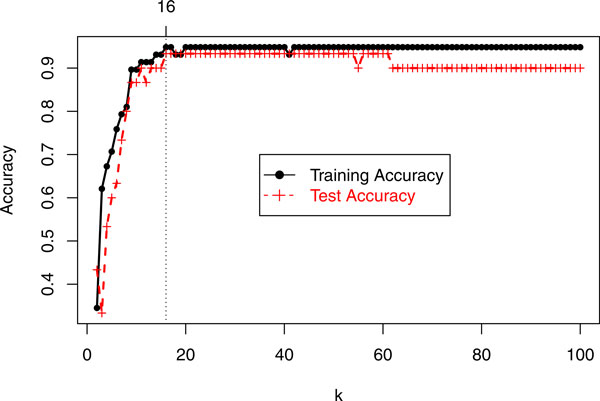
**Accuracy of TS_k _for training and test data from DLBCL cancer (Alizadeh et al., 2000)**.

As an illustration, Figure 1 shows the the accuracy of the training and test data for *k *= *2*,..., *100 *for the 6-class DLBCL cancer microarray data [17]. It can be seen that the training accuracy reached maximum when *k *= *16*. So the selected marker set is TS_16_.

Our experience suggests that it is sufficient to set the upper bound B to be 50 if the total number of classes M ≤ 4, 100 if 5 ≤ M ≤ 8, and 150 if M ≥ 9.

### Prediction with TSG classifier

To predict the class information for each sample in the test data, we use the selected marker set and calculate the scores of this sample belonging to each class. A large value for a class suggests that putting this sample in that class helps to increase the separation of different classes. The predicted class is set to be the one that has the largest score. In particular, suppose the selected marker set consists of markers *m_1_, m_2_,..., m_k_*, the training data is Ω_tr_, and the sample to be predicted is *x*_new_. Let${\chi^{2}}_{i_{1}...i_{k}|C_{i}}$be the Chisquare statistic value when we put the sample in class *C_i_, i = 1,..., M*. There are M Chisquare values. We assign the sample to the class with the largest Chisquare value:

Class of $x_{\text{new}}=\text{arg}\underset{i=1,...,M}{\text{max}}{\chi^{2}}_{i_{1}...i_{k}{|}_{}C_{i}}$.

If multiple classes reach the same maximum Chisquare value, we further calculate the LOOCV accuracy for these classes. The final prediction is based on which class achieves the highest LOOCV accuracy.

## Results and discussion

### Microarray data and method of comparison

The performance of the proposed TSG marker selection and classifier is evaluated on both binary and multi-class microarray expression data. We consider the 19 datasets that were used for evaluation of TSP, k-TSP and their multi-class version classifiers in Tan et al. 2005. There are 9 binary and 10 multi-class datasets. These datasets are related to human cancers including colon cancer, leukemia, central nervous system, diffuse large B-cell lymphoma, breast cancer, lung cancer, and prostate cancer. The reference, sample size, number of genes in each dataset, and the number of samples in each class are summarized in Tables 3 and 4. The number of classes ranges from 2 to 14. The number of markers ranges from 2000 to 16063. Average number of samples per class ranges from 13 to 140. The ratio between the number of samples per class and the number of markers ranges from 0.000845 to 0.0155.

**Table 3:** Binary class gene expression datasets

Dataset	Platform	No. of Genes	No. of samples in class I	No. of samples in class II	Source
Colon	cDNA	2000	40(T)	22(N)	[18]
Leukemia	Affy	7129	25(AML)	47(ALL)	[19]
CNS	Affy	7129	25(C)	9(D)	[20]
DLBCL	Affy	7129	58(D)	19(F)	[21]
Lung	Affy	12533	150(A)	31(M)	[22]
Prostate1	Affy	12600	52(T)	50(N)	[23]
Prostate2	Affy	12625	38(T)	50(N)	[24]
Prostate3	Affy	12626	24(T)	9(N)	[25]
GCM	Affy	16063	190(C)	90(N)	[26]

**Table 4:** Multi-class gene expression datasets

Dataset	Platform	No. of Classes	No. of Genes	No. of samples in training	No. of samples in test	Source
Leukemia1	Affy	3	7129	38	34	[19]
Lung1	Affy	3	7129	64	32	[27]
Leukemia2	Affy	3	12582	57	15	[28]
SRBCT	cDNA	4	2308	63	20	[29]
Breast	Affy	5	9216	54	30	[30]
Lung2	Affy	5	12600	136	67	[31]
DLBCL	cDNA	6	4026	58	30	[17]
Leukemia3	Affy	7	12558	215	112	[32]
Cancers	Affy	11	12533	100	74	[33]
GCM	Affy	14	16063	144	46	[26]

First, we consider comparison of TSG and k-TSP classifiers for binary datasets based on 5-fold cross validation. The subjects in each class are randomly partitioned into 5 parts, 4 of which form the training data and the rest of the subjects constitute the test data. The feature selection and modeling were conducted on the training data and prediction for each subject in the test data was given. For the results to be comparable to the TSP family classifiers, we also follow the same comparison methods as in Tan et al. [3]. In particular, we perform LOOCV for binary datasets and perform independent test for multi-class datasets. In the LOOCV, each sample is taken out and the remaining N-1 samples are used to train the classifier, which is then used to predict the class label of the leave-out sample. The LOOCV accuracy is the proportion of correctly classified samples. Each of the multi-class datasets is partitioned into training and test data. We follow exactly the same partition scheme as in Tan et al. [3].

Since an objective of this research is to improve the TSP family classifiers, we present the percent of increase in classification accuracy in barplots. The percent of increase for TSG over TSP is defined as (accuracy of TSG - accuracy of TSP)/accuracy of TSP x 100%.

The percent of increase for any two classifiers are similarly defined. As TSP classifier uses two genes, k-TSP and TSG use at least two genes, we are particularly interested in comparing the improvement in accuracy for TSG over TSP and k-TSP over TSP in binary classifications. Similarly, in the multi-class cases, we are interested in comparing the increase in accuracy for TSG over HC-TSP and HC-k-TSP over HC-TSP.

For reference, we also include the classification accuracy of decision trees (DT), Naive Bayes (NB), k-nearest neighbor (k-NN), Support Vector Machines (SVM) and prediction analysis of microarrays (PAM) in our comparison tables when they are available from the literature. These results were reported in Tan et al. [3] for leave-one-out cross validation for binary data and independent test for multiclass data. We include them only for convenience of discussion. DT and PAM have feature selection function while NB, k-NN and SVM perform classification using the entire set of features. Since DT, k-NN, and NB in general have lower accuracy than the other classifiers, we focus our discussion on other classifiers.

### Accuracy for binary cancer data

In this section we present the comparison of the TSG marker selection algorithm with other algorithms using 9 benchmark binary class cancer expression datasets. These data have been analyzed extensively by many authors with wrapper, filtering and ensemble methods.

#### Comparison of TSG and k-TSP classifiers based on 5-fold cross validation

Here we present our comparison of TSG and k-TSP classifiers based on 5-fold cross validation. For each binary cancer dataset, we randomly split the subjects in each class into 5 parts. One part will be left out as the test data and the remaining 4 parts are used as the training data. We then train the TSG, k-TSP and TSP classifiers using the training data to select features and build models. TSP is included for reference purpose since both TSG and k-TSP extend the TSP classifier. The resulting features and models are further used to predict the class of each subject in the test data. Each of the 5 parts in turn serves as the test data. We record the accuracy of the prediction calculated as the proportion of correctly classified subjects among all collected subjects. The procedure is repeated 10 times. The average and standard error of the accuracy are reported in Table 5.

**Table 5:** Average and standard error of accuracy from 5-fold cross validation based on 10 runs.

	TSG		k-TSP		TSP	
	
Dataset	Average	Standard error	Average	Standard error	Average	Standard error
CNS	81.1	1.61	77.0	1.48	67.0	2.48
Colon	84.8	0.85	87.4	0.31	86.7	1.85
DLBCL	95.2	0.69	94.6	0.88	92.9	2.25
GCM	81.7	0.66	81.9	0.41	76.0	1.04
Leukemia	93.7	0.75	91.8	1.13	89.4	1.56
Lung	100	0	98.6	0.15	96.3	0.69
Prostate1	90.2	0.72	91	0.75	89.3	1.27
Prostate2	74.4	0.6	77.9	0.69	70.4	2.25
Prostate3	100	0	95.4	0.79	95.1	1.44
Average	89.0		88.4		84.8	

In five of the nine datasets (CNS, DLBCL, Leukemia, Lung, Prostate3), TSG has slightly better performance than k-TSP. In the remaining four datasets, k-TSP has slightly better performance. On average, TSG and k-TSP have comparable 5-fold cross validation performance. The k-TSP improvement over TSP is also marginal in this 5-fold cross validation setting for most of the datasets.

#### LOOCV accuracy

Among the LOOCV accuracy reported in the literature, we find that TSP, k-TSP, PAM and SVM are often the top performing classifiers.

The LOOCV accuracy of the proposed TSG and aforementioned competing classifiers for the 9 datasets are presented in Table 6. In terms of accuracy, TSG consistently gives the best performance for all but the leukemia dataset, for which NB yields an accuracy of 100% while TSG gives 98.61%. For the CNS data, TSG and k-TSP have tied performance of 97% that is much better than the rest of the classifiers (all below 83%). For the prostate 1 data, TSG and TSP have equally best performance. For the prostate 3 data, TSG and SVM both have 100% accuracy.

**Table 6:** LOOCV accuracy and the number of genes used in classifiers (in parenthesis) for binary class expression datasets

Method	Colon	Leuk	CNS	DLBCL	Lung	Pros1	Pros2	Pros3	GCM	Aver
TSG^†^	**93.55** (2)	98.61 (2)	**97.06** (2)	**98.7** (2)	**100** (2)	**95.1** (2)	**86.36** (10)	**100** (2)	87.5 (7)	**95.21**
TSP*	91.10 (2)	93.80 (2)	77.90 (2)	98.10 (2)	98.30 (2)	**95.10** (2)	67.60 (2)	97.00 (2)	75.40 (2)	88.26
k-TSP*	90.30 (2)	95.83 (18)	97.10** (10)	97.40 (2)	98.90 (10)	91.18 (2)	75.00 (18)	97.00 (2)	85.40 (10)	92.01
DT*	77.42 (3)	73.61 (2)	67.65 (2)	80.52 (3)	96.13 (3)	87.25 (4)	64.77 (4)	84.85 (1)	77.86 (14)	78.90
NB*^‡^	56.45	**100**	82.35	80.52	97.79	62.75	73.86	90.91	84.29	80.99
k-NN*^‡^	74.19	84.72	82.35	89.61	98.34	74.51	73.86	93.94	86.79	84.26
SVM*^‡^	82.26	98.61	82.35	97.40	99.45	91.18	76.14	**100**	**93.21**	91.18
PAM*	89.52 (15)	94.03 (2296)	82.35 (4)	85.45 (17)	97.90 (9)	90.89 (47)	81.25 (13)	94.24 (701)	82.32 (47)	88.66

*Results reported in Tan et al. [3]^†^Results obtained with our method (TSG) ^‡^NB, k-NN, SVM used entire set of genes

**The 97.10 reported in Tan et al. [3] may be a result of rounding 97.06, which is the accuracy of correctly classifying 33 of the 34 samples.

**Figure 2:**
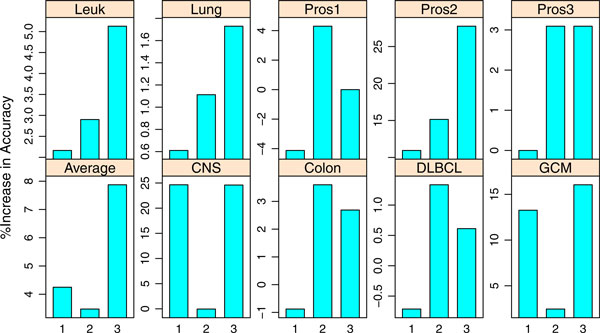
**Percent of improvement in accuracy for TSG over TSP family classifiers in binary classification**. 1: k-TSP improve upon TSP, 2:TSG improve upon k-TSP, 3: TSG improve upon TSP.

To assess how much improvement the TSG classifier achieves by considering arbitrary number of genes instead of only top scoring pairs as in TSP and k-TSP, we give the percent of increase in LOOCV accuracy in Figure 2. The first bar in each panel is the percent of improvement in accuracy for k-TSP over TSP classifier; the second gives that for TSG over k-TSP and the third gives that for TSG over TSP. Comparing the bar heights of the first and third bars in each panel gives us an idea of whether k-TSP and TSG improve TSP with similar performance. The second bar in each panel tells how much improvement TSG achieves over k-TSP. For the CNS data, TSG and k-TSP have same improvement over TSP. The much taller heights for the third bar in all panels except for the CNS data suggest that TSG gained much more accuracy than k-TSP. There are two reasons for this observed accuracy gain: (1) the set of informative genes could be odd numbered but k-TSP can only use even number of genes; (2) selection of additional genes after the top pair by the TSG classifier considers the joint effect of all selected genes on differentiating the cancer classes whereas the k-TSP classifier only consider the marginal effect of pairs. Without considering the joint effect, collection of top scoring pairs in k-TSP could easily accumulate redundant genes. So TSG naturally gives better performance than k-TSP.

**Figure 3:**
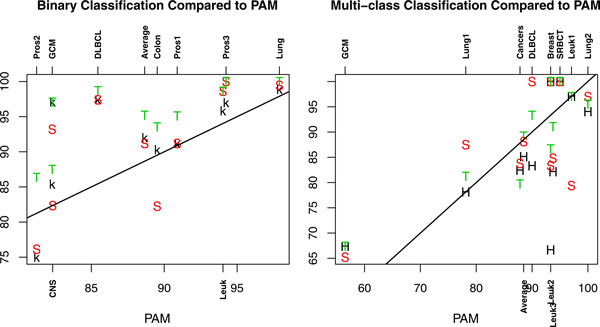
**The accuracy of TSG, k-TSP, HC-k-TSP, and SVM compared to PAM**. Left: LOOCV accuracy of TSG (T), k-TSP (k) and SVM (S) compared to PAM in binary datasets; Right: Accuracy for independent test samples for TSG (T), HC-k-TSP (H), and SVM (S) compared to PAM in multi-class datasets. The straight lines in the plots have slope 1 and intercept 0 such that points on the lines have equal horizontal and vertical values.

To ease the discussion about how the performance of TSG, k-TSP, HC-k-TSP, SVM, and PAM classifiers compare to each other, we plot the LOOCV accuracy of TSG, k-TSP, and SVM relative to that of PAM in the left panel of Figure 3. It can be seen that TSG, k-TSP, and SVM are in general better than PAM for binary data since most of the accuracy values for TSG, k-TSP, and SVM are above the straight line. The performance of TSG is consistently the best as its values are highest for all datasets. SVM has similar accuracy as k-TSP in four datasets. SVM is worse than k-TSP clearly in two datasets (Colon and CNS) and k-TSP is obviously not as accurate as SVM in the Leuk, GCM and Pros3 datasets. On average performance, k-TSP and SVM are comparable in binary classifications with the 9 datasets. In summary, the TSG classifier outperforms k-TSP and SVM in LOOCV accuracy for the 9 binary classification problems, the latter two have comparable performance and are both better than PAM in accuracy.

### Accuracy of independent test for multi-class cancer data

For the multi-class datasets, the accuracy of classifiers on independent test data is presented in Table 7.

**Table 7:** Accuracy of classifiers and the number of genes used in classifiers (in parenthesis) for the independent test set for multi-class expression datasets

Method	Leuk1	Lung1	Leuk2	SRBCT	Breast	Lung2	DLBCL	Leuk3	Cancers	GCM	Aver
TSG^†^	**97.06** (6)	81.25 (20)	**100** (44)	**100** (13)	86.67 (63)	95.52 (60)	93.33 (16)	91.07 (95)	79.73 (81)	**67.39** (112)	**89.20**
HC-TSP*	**97.06** (4)	71.88 (4)	80 (4)	95 (6)	66.67 (8)	83.58 (8)	83.33 (10)	77.68 (12)	74.32 (20)	52.17 (26)	78.17
HC-k-TSP*	**97.06** (36)	78.13 (20)	**100** (24)	**100** (30)	66.67 (24)	94.03 (28)	83.33 (46)	82.14 (64)	82.43 (128)	**67.39** (134)	85.12
DT*	85.29 (2)	78.13 (4)	80 (2)	75 (3)	73.33 (4)	88.06 (5)	86.67 (5)	75.89 (16)	68.92 (10)	52.17 (18)	76.35
NB^‡^*	85.29	81.25	100	60	66.67	88.06	86.67	32.14	79.73	52.17	73.2
k-NN^‡^*	67.65	75	86.67	30	63.33	88.06	93.33	75.89	64.86	34.78	67.96
PAM*	**97.06** (44)	78.13 (13)	93.33 (62)	95 (285)	**93.33** (4822)	**100** (614)	90 (3949)	**93.75** (3338)	**87.84** (2008)	56.52 (1253)	88.5
1-vs-1-SVM^‡^*	79.41	**87.5**	**100**	**100**	83.33	97.01	**100**	84.82	83.78	65.22	88.11

*Results reported in Tan et al. [3]^†^Results obtained with our method (TSG) ^‡^: NB, k-NN, 1-vs-1-SVM used entire set of genes in classification. That is, 7129, 7129, 12582, 2308, 9216, 12600, 4026, 12558, 12533, 16063 for the ten data sets respectively.

**Figure 4:**
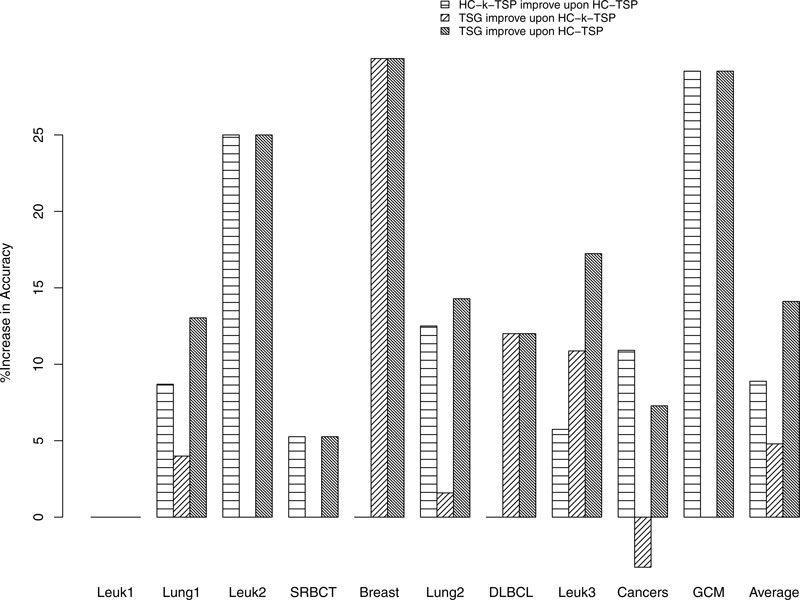
**Percent of improvement in accuracy for TSG over TSP family classifiers in multi-class classification**.

The percent of increase in accuracy for HC-k-TSP and TSG over HC-TSP is shown in Figure 4. The similar bar heights for the first and third bars in Leuk1, Leuk2, SRBCT, and GCM datasets indicate that HC-k-TSP and TSG improve upon TSP with similar amount for these datasets. For the Breast, DLBCL, Leuk3 datasets, TSG achieved a lot more accuracy gain over HC-TSP than HC-k-TSP. For these three datasets, the gain of accuracy of TSG over HC-k-TSP is 30%, 12%, and 10.87% respectively. For Lung1, Lung2 and on average, TSG also has better accuracy than HC-k-TSP but the gain of TSG over HC-k-TSP is not more than 5%. For the cancers dataset, TSG lost 3.27% accuracy than HC-k-TSP. In summary, TSG gains more accuracy than HC-k-TSP in all except for one dataset. We remark that there are three schemes of extending binary classifier TSP to multi-class classifiers (1-vs-1, 1-vs-others, and hierarchical classification schemes). The HC-k-TSP and HC-TSP results reported in Tan et al. [3] are with the hierarchical scheme that performs best out of all three schemes. Therefore, TSG classifier has even better performance than the 1-vs-1 and 1-vs-others multi-class extensions of TSP family classifiers.

The accuracy based on independent test samples for TSG, HC-k-TSP, and SVM relative to the accuracy of PAM is presented in the right panel of Figure 3. For half of the datasets SVM has better performance than PAM and for the other half of the datasets, PAM performs better than SVM in accuracy. So on average, SVM and PAM are comparable. The accuracy of HC-k-TSP in majority of datasets is below that of PAM. On average, HC-k-TSP has accuracy slightly lower than PAM. TSG has better accuracy than PAM in five of ten datasets, and TSG is not as accurate as PAM in four datasets. On average, TSG, PAM and SVM have comparable performance with TSG only slightly better. In summary, for multi-class problems, TSG, SVM and PAM are better than HC-k-TSP in accuracy.

### The number of genes used in classifiers

**Figure 5:**
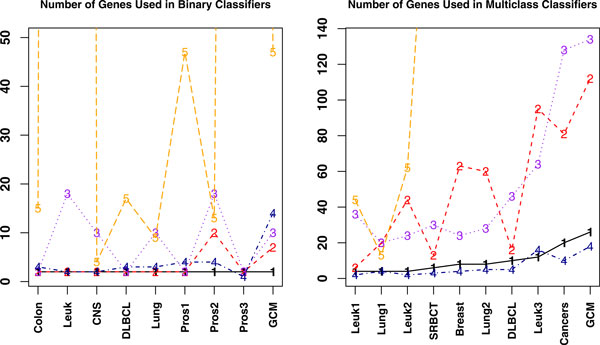
**Plot of the number of genes for TSP(or HC-TSP), TSG, k-TSP(or HC-k-TSP), DT, and PAM**. Labels 1,..., 5 in plots represent: 1: TSP in binary and HC-TSP in multi-class cases; 2: TSG; 3: k-TSP in binary and HC-k-TSP in multi-class cases; 4: DT; 5: PAM. The number of genes used in PAM is not shown in the graph if they are beyond the upper limit of the vertical axis. The numbers not shown for PAM include 2296 and 701 for Leuk and Pros3 data respectively on binary cases, and 285, 4822, 614, 3949, 3338, 2008, 1253 genes for SRBCT, Breast, Lung2, DLBCL, Leuk3, Cancers, and GCM data respectively in multi-class cases.

For comparison of classifiers with finite number of samples, the number of genes used by each classifier is an important factor. Classifiers using more genes tend to overfit the data. Hence classifiers with small number of genes are preferred. Since SVM, k-NN, and NB use the entire set of genes in their classification algorithm, we eliminate them from further discuss on number of genes used and restrict the rest of the discussion in this subsection to TSP, HC-TSP, DT, k-TSP, HC-k-TSP, TSG, and PAM. For these classifiers, we plot the number of genes used in each dataset for each classifier. For quite many datasets, PAM used hundreds or thousands of genes in the final classifiers. So we set the upper limit of the vertical axis in Figure 5 to be 50 in binary cases and 140 in multi-class cases so that the numbers used by other classifiers can be seen. The numbers for the same classifier under different datasets are connected for convenience of viewing.

It can be seen from Figure 5 that TSP classifier has the lowest number of genes in binary data classification. PAM used much more number of genes than other classifiers. The classifier that has the second smallest number of genes in binary data classification is TSG, followed by DT that uses the third smallest number of genes. The k-TSP classifier in general used more number of genes than TSP, TSG, and DT.

For multi-class data, DT uses the least number of genes followed by HC-TSP in the second place. However, as discussed in Section 3.1, DT and HC-TSP are not as accurate as SVM, PAM, HC-k-TSP, and TSG. In 4 out of 10 datasets TSG used more genes than HC-k-TSP and in 5 datasets TSG used fewer genes than HC-k-TSP. On average across all 10 datasets, TSG uses 51 genes and HC-k-TSP uses 53.4 genes. PAM consistently uses a lot more genes than other classifiers except in Lung1 data. In fact, the number of genes used by PAM is in the magnitude of thousands in order to reach comparable accuracy as TSG. Recall that on average TSG, PAM and SVM have comparable accuracy for independent test data and HC-k-TSP has lower performance. Now combining the accuracy and the number of genes used, TSG outperforms the rest in that it uses smaller number of genes to reach high accuracy. Smaller number of genes makes it feasible to perform follow-up studies and further experimental verification after the informative genes selection.

### Interpretation of the TSG classifier

The TSG classifier has an easy interpretation. Recall that the main rule to classify a sample is based on which class gives the highest Chisquare statistic value when the sample is assigned to that class (see section 2.3). Note that there is a one to one correspondence between the Chisquare statistic values and the degree of departure from independence between the class label and the column variables in Table 2. The bigger the Chisquare statistic, the further the departure is from independence and vice versa. So the class label assignment tries to maximize the dependence between the classes and the selected variables for a given set of training data and a test sample.

The TSG classifier has the same interpretation as the TSP classifier. In particular, suppose the expression values for these two genes are E_1 _and E_2 _for a sample, the prediction of the class label of this sample depends on whether E_1 _< E_2_. For Leuk, CNS, and pros3 data, TSG achieved accuracy 98.61, 97.06, and 100 for these three datasets respectively using 2 genes. Also using 2 genes, TSP only achieved 93.80, 77.90, 97.00 in LOOCV accuracy for these three datasets. Therefore, TSG finds genes that are even more informative than TSP classifier in these three datasets. When the selected informative genes have more than 2 genes, then the class prediction of a sample depends on all pairwise comparisons of the expression values $E_{i_{1}},\cdots \;,E_{i_{k}}$ for the selected genes i_1_, i_2_,..., i_k _from this sample, i.e., which of the inequalities $E_{i_{1}}<E_{i_{2}}$, $E_{i_{1}}\geq E_{i_{2}}$,......, $E_{i_{k-1}}<E_{i_{k}}$, $E_{i_{k-1}}\geq E_{i_{k}}$ are true. Due to the similar interpretation to the TSP family classifiers, we do not reiterate for TSG and refer the readers to Tan et al. [3] for details.

## Conclusions

In this article, we presented the TSG classifier, an improved version of TSP family classifiers for both binary and multi-class cancer classification. The TSP family classifiers only consider even number of genes and the gene selection process is based on the marginal comparison of pairwise expression values without honoring the fact that some of the marginally important genes may have similar effects as others and therefore could be redundant. We solved aforementioned shortcomings of TSP family classifiers by allowing both even and odd number of genes through newly defined score functions and a new selection algorithm. After some genes have been selected, our gene selection process assesses the importance of additional genes by considering the overall contribution of all the genes included in the informative set. As the joint effects of multiple genes are evaluated together, we expect that the final list of genes selected by TSG is more parsimonious than k-TSP and HC-k-TSP classifiers.

The TSG classifier is in a simple unified form for both binary and multi-class cases. This is different from the TSP family classifiers in that three binary to multi-class extension schemes (1-vs-1, 1-vs-others, hierarchical classification) lead to three different classifiers. As reported in Tan et al [3], the hierarchical classification scheme extension HC-TSP and HC-k-TSP perform the best out of the three schemes. Our TSG classifier is in a single form and in general has equal performance or outperforms k-TSP, HC-TSP and HC-k-TSP in the 19 datasets in terms of accuracy and number of genes used. We also compared the performance of TSG with PAM and SVM. In binary classification problems, TSG has better LOOCV accuracy than PAM and SVM; in multi-class problems, TSG, PAM, and SVM give comparable accuracy for independent test data. All three classifiers are more accurate than TSP family classifiers. In terms of the number of genes used, TSG clearly uses much less number of genes than PAM and SVM. PAM often selects thousands of genes in its final classifier and SVM uses the entire set of genes.

An obvious advantage of the TSG as well as the TSP family classifiers is that they are based on the simple pairwise comparisons of expression values between genes from the same sample. Such comparison is robust to monotone transformation and eliminates the concern about variations among different patients, platforms, or bias from preprocessing different samples. Therefore, we expect that the results from TSG are more reliable and robust compared to many other methods that pool the data from different samples to filter genes.

## List of abbreviations used

TSP: top scoring pair; k-TSP: k top scoring pairs; HC-TSP: multi-class extension of TSP with hierarchical classification scheme; HC-k-TSP: multi-class extension of k-TSP with hierarchical classification scheme; SVM: Support Vector Machine classification; PAM: Prediction Analysis of Microarray; LOOCV: leave-one-out cross validation.

## Competing interests

The authors declare that they have no competing interests.

## Authors' contributions

ZY and HZ developed the algorithm; HZ performed gene selection and classification for the microarray data; HW conducted some of the comparisons, summarized the results and drafted the manuscript; HZ and ZD designed the software; ZY and MC provided discussion and revised the manuscript for this study. All authors have approved the final version of the manuscript.
